# Joubert Syndrome and related disorders

**DOI:** 10.1186/1750-1172-5-20

**Published:** 2010-07-08

**Authors:** Francesco Brancati, Bruno Dallapiccola, Enza Maria Valente

**Affiliations:** 1 Mendel Laboratory, Casa Sollievo della Sofferenza Hospital, IRCCS, San Giovanni Rotondo, Italy; 2 Department of Biomedical Sciences, Ce.S.I. Aging Research Center, Gabriele d'Annunzio University Foundation, Chieti, Italy; 3 Bambino Gesù Children Hospital, IRCCS, Rome, Italy; 4 Department of Medical and Surgical Pediatric Sciences, University of Messina, Messina, Italy

## Abstract

Joubert syndrome (JS) and related disorders (JSRD) are a group of developmental delay/multiple congenital anomalies syndromes in which the obligatory hallmark is the molar tooth sign (MTS), a complex midbrain-hindbrain malformation visible on brain imaging, first recognized in JS. Estimates of the incidence of JSRD range between 1/80,000 and 1/100,000 live births, although these figures may represent an underestimate. The neurological features of JSRD include hypotonia, ataxia, developmental delay, intellectual disability, abnormal eye movements, and neonatal breathing dysregulation. These may be associated with multiorgan involvement, mainly retinal dystrophy, nephronophthisis, hepatic fibrosis and polydactyly, with both inter- and intra-familial variability. JSRD are classified in six phenotypic subgroups: Pure JS; JS with ocular defect; JS with renal defect; JS with oculorenal defects; JS with hepatic defect; JS with orofaciodigital defects. With the exception of rare X-linked recessive cases, JSRD follow autosomal recessive inheritance and are genetically heterogeneous. Ten causative genes have been identified to date, all encoding for proteins of the primary cilium or the centrosome, making JSRD part of an expanding group of diseases called "ciliopathies". Mutational analysis of causative genes is available in few laboratories worldwide on a diagnostic or research basis. Differential diagnosis must consider in particular the other ciliopathies (such as nephronophthisis and Senior-Loken syndrome), distinct cerebellar and brainstem congenital defects and disorders with cerebro-oculo-renal manifestations. Recurrence risk is 25% in most families, although X-linked inheritance should also be considered. The identification of the molecular defect in couples at risk allows early prenatal genetic testing, whereas fetal brain neuroimaging may remain uninformative until the end of the second trimester of pregnancy. Detection of the MTS should be followed by a diagnostic protocol to assess multiorgan involvement. Optimal management requires a multidisciplinary approach, with particular attention to respiratory and feeding problems in neonates and infants. Cognitive and behavioral assessments are also recommended to provide young patients with adequate neuropsychological support and rehabilitation. After the first months of life, global prognosis varies considerably among JSRD subgroups, depending on the extent and severity of organ involvement.

## Disease name and synonyms

The term Joubert Syndrome and Related Disorders (JSRD) has been recently adopted to describe all disorders presenting the "molar tooth sign" (MTS) on brain imaging. Thus, JSRD include Joubert syndrome (JS, also known as Joubert-Boltshauser syndrome [OMIM#213300]), as well as any related condition showing the MTS, such as the cerebello-oculo-renal syndrome, Dekaban-Arima syndrome [OMIM%243910], COACH syndrome [OMIM216360], Varadi-Papp syndrome (or Orofaciodigital type VI, [OMIM%277170]), Malta syndrome and a minority of cases with Senior-Loken syndrome [OMIM#266900].

## Definition and diagnostic criteria

Joubert syndrome (JS) was originally described in 1968 in four siblings with agenesis of the cerebellar vermis presenting episodic hyperpnoea, abnormal eye movements, ataxia and intellectual disability [1]. Several years later, a pathognomonic midbrain-hindbrain malformation, the "molar tooth sign" (MTS), was detected first in JS [2], and then in several other conditions previously considered as distinct entities [3] (see section "Disease name and synonyms"). The term "Joubert Syndrome and Related Disorders" (JSRD) was then coined to group all conditions sharing the MTS [4], and this neuroradiological sign now represents the mandatory criterion to diagnose JSRD.

**Figure 1:**
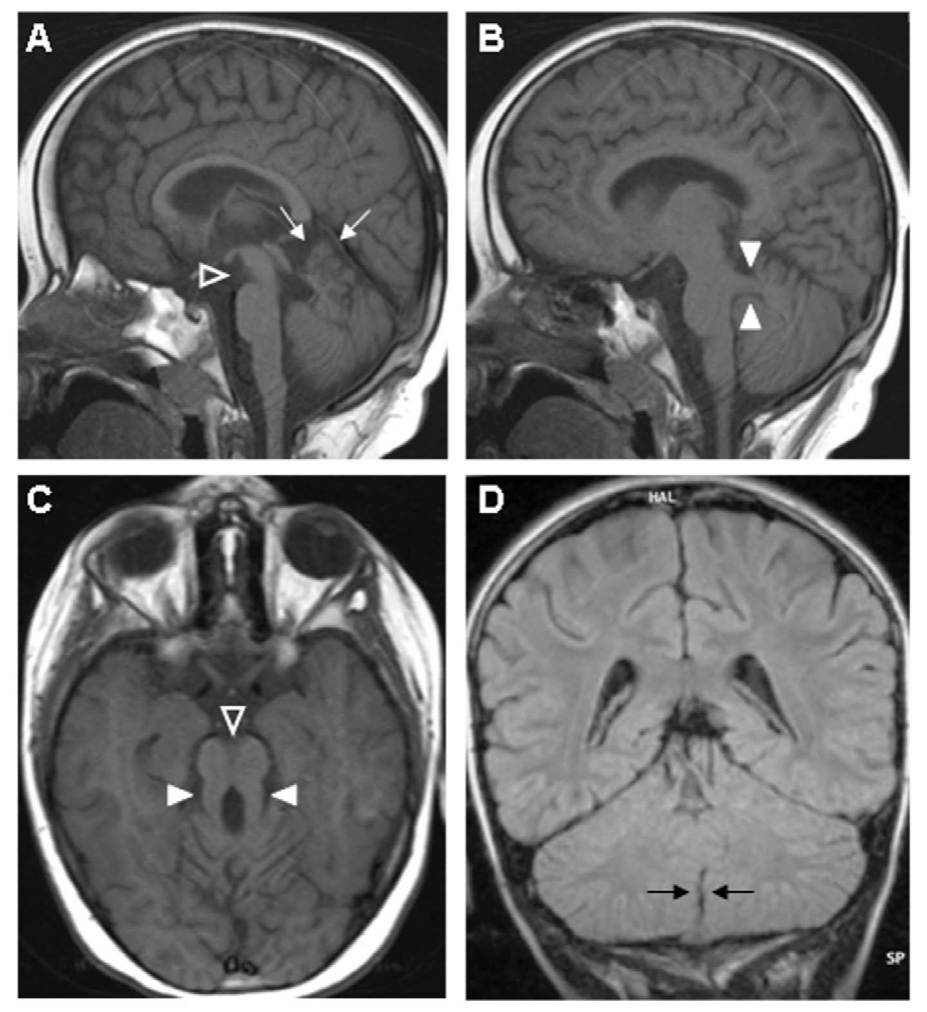
**Brain MRI sections in patients with JSRD**. A) mid-sagittal T1-weighted image shows a thin midbrain with corresponding enlargement of the interpeduncular fossa (open arrowhead). There is concurrent superior vermian dysplasia (thin arrows); B) parasagittal T1-weighted image shows thickened and maloriented superior cerebellar peduncle (thick arrowheads); C) axial T1-weighted image confirms the deepened interpeduncular fossa (open arrowhead) and abnormal superior cerebellar peduncles (thick arrowheads), comprising the "molar tooth sign"; D) coronal FLAIR image shows midline cerebellar cleft (black arrows) indicating agenesis of the inferior vermis.

The MTS results from hypo-dysplasia of the cerebellar vermis, abnormally deep interpeduncular fossa at the level of the isthmus and upper pons, and horizontalized, thickened and elongated superior cerebellar peduncles [5] (Figure 1). At the pathological level, these abnormalities correspond to a picture of severe hypo-dysplasia of the cerebellar vermis with midline clefting, fragmentation of cerebellar nuclei and heterotopia of Purkinje-like neurons, along with dysplasia of pontine and medullary structures such as the basis pontis, reticular formation, inferior olivary, dorsal column and solitary tract nuclei. Moreover, typical findings are represented by the lack of decussation both of the superior cerebellar peduncles and of the corticospinal tracts at the medullary pyramids [1,6].

JSRD are clinically heterogeneous and combine neurological signs with variable multiorgan involvement, mainly of the retina, kidneys, liver and skeleton. This marked pleiotropism can probably be explained by the genetic basis of these syndromes. Ten causative genes have been identified to date: JBTS1/*INPP5E *[OMIM*613037], JBTS2/*TMEM216 *[OMIM*613277], JBTS3/*AHI1 *[OMIM*608894], JBTS4/*NPHP1 *[OMIM*607100], JBTS5/*CEP290 *[OMIM*610142], JBTS6/*TMEM67 *[OMIM*609884], JBTS7/*RPGRIP1L *[OMIM*610937], JBTS8/*ARL13B *[OMIM*608922], JBTS9/*CC2D2A *[OMIM*612013], JBTS10/*OFD1 *[OMIM*300170]. All these genes encode for proteins of the primary cilium, including JSRD in the group of "ciliopathies". Primary cilia are known to play key roles in the development and functioning of several cell types, including retinal photoreceptors, neurons, kidney tubules and bile ducts [7,8]. In the developing cerebellum and brainstem, these organelles regulate major signal transduction pathways, and have been implicated both in neuronal cell proliferation and axonal migration [9].

JSRD present genetic and clinical overlap with other ciliopathies, in particular with Meckel syndrome, a lethal malformative condition characterised by encephalocele, other posterior fossa anomalies, ductal plate malformation of the liver and polycystic kidneys. JSRD and Meckel syndrome phenotypes may represent the two ends of a continuum and, indeed, mutations in at least five genes (*TMEM216*, *CEP290*, *TMEM67*, *RPGRIP1L *and *CC2D2A*) cause both disorders, with distinct mutation types variably correlating with the phenotypic severity [10-15]. Interestingly, preliminary genotype-phenotype correlates indicate that Meckel syndrome fetuses nearly invariably harbor truncating mutations, while at least one hypomorphic (e.g. missense) mutation is found in almost all JSRD cases [11,13,14].

## Epidemiology

Although the incidence of JSRD has not been precisely determined, it may range between 1/80,000 and 1/100,000 live births, but may be underestimated [16,17].

## Clinical description

### Neurological features

The cardinal neurological features of JSRD are hypotonia evolving into ataxia and developmental delay, often associated with intellectual disability, altered respiratory pattern in the neonatal period and abnormal ocular movements [1,18-20].

Early hypotonia is observed in nearly all JSRD patients and can be recognized in the neonatal period or in infancy. Although hypotonia represents an unspecific finding of many neuropediatric disorders, its association with other peculiar features such as an irregular breathing pattern and altered eye movements should suggest the diagnosis of JSRD and prompt the clinician to request a brain magnetic resonance imaging. Patients often develop ataxia with broad-based gait in the first years of independent ambulation. These balance difficulties have been extensively reviewed [2] and, although non-specific, represent a frequent finding of JSRD. In some cases, tremor has been reported [20].

The typical respiratory abnormalities are represented by short alternate episodes of apnea and hyperpnea or episodic hyperpnea alone, which tend to occur shortly after birth, intensify with emotional stress and progressively improve with age, usually disappearing around the sixth month of life. Their severity can range from occasional short-lasting episodes manifesting every few days to extremely frequent (up to several per day) and prolonged attacks of apnea, requesting intensive care management and assisted ventilation [1,21].

Abnormal eye movements also represent a recurrent feature in JSRD. Oculomotor apraxia is one of the most characteristic and frequent abnormalities, that manifests with the inability to follow objects visually with compensatory head movements, decreased smooth pursuit, and cancellation of the vestibulo-ocular reflex. Primary position nystagmus is also common, while occasionally associated features include strabismus and ptosis. Such manifestations are present independently from the specific defects of the eyes (see "Ocular features"), and relate to the underlying midbrain-hindbrain malformation [2,21-23].

Developmental abilities, in particular language and motor skills, are delayed in all JSRD patients, with variable degrees of severity [22,24-26]. Mild to severe intellectual disability is common, with several patients being able to attend special schools, learn specific job skills and work in protected conditions [27]. However, it must be stressed that intellectual deficit is not a mandatory feature of JSRD and exceptional cases may have borderline or even normal intellect. Siblings discordant for this feature have been recently reported, further emphasizing the difficulty to predict the neurological outcome in infants [28,29].

A wide range of central nervous system (CNS) malformations are described in association to the MTS that can dramatically affect the clinical outcome and prognosis of patients with JSRD. These include hydrocephalus, cystic enlargement of the posterior fossa, abnormalities of the corpus callosum, white matter cysts, hypothalamic hamartoma, and absence of the pituitary gland [30-38]. Abnormal migration defects, mainly periventricular nodular heterotopia, and cortical organization defects such as polymicrogyria have also been reported [39,40]. Patients with these malformations present a higher incidence of epilepsy, which is otherwise a rare feature of JSRD [32,39]. Finally, a small number of cases present with occipital (meningo) encephalocele of variable severity [1,34,41].

### Ocular features

The retina is one of the organs most frequently involved in JSRD, mostly in the form of retinal dystrophy, due to progressive degeneration of photoreceptor cells. The clinical spectrum can range from congenital retinal blindness (also known as Leber congenital amaurosis - LCA), to retinal dystrophy characterized by a progressive course and variably conserved vision [2,42-45]. The diagnosis is based on the findings of reduced visual acuity, associated with abnormal *fundus oculi *and/or electroretinogram [3]. The association of LCA with nephronophthisis (NPH) defines the Senior-Loken syndrome that, in a subset of cases, also displays the MTS [3].

Colobomas can be unilateral or bilateral, and mostly affect the posterior segment of the eye. Such defects originate from a failure in fetal fissure closure, which results in colobomas involving the retinal pigmented epithelium, the neurosensory retina or the choroids [46]. Iris colobomas have been reported in few cases [3]. Colobomas represent a rare cause of visual impairment in all JSRD clinical subgroups, although their frequency reaches over 30% in the subgroup of JS with hepatic defect [47] (see "Classification of JSRD").

### Renal features

Renal disease affects approximately 25% of patients with JSRD, presenting in most cases as NPH. This is a structural tubulo-interstitial disorder characterized by irregular, thickened basal membrane of the tubular epithelium and progressive interstitial fibrosis, associated with small cysts at the cortico-medullary junction. Juvenile NPH may remain asymptomatic for several years or present with subtle and often unrecognized signs such as polyuria and polydypsia, until acute or chronic renal insufficiency manifests in the late first or early second decade of life. End stage renal failure is usually reached by the end of the second decade, requiring dialysis or kidney transplantation. An infantile variant of NPH manifests within the first years of life, with a more rapid and severe course [48].

The association of MTS with cystic dysplastic kidney (CDK), characterized by abnormal metanephric development with macroscopically large kidneys and prominent cysts, prompted some authors to delineate a novel clinical entity termed Dekaban-Arima syndrome [OMIM 243910] [18,49,50]. However, a recent re-examination of some of the originally reported patients showed histological abnormalities more typical of NPH than of CDK, challenging the existence of Dekaban-Arima syndrome and suggesting that all JSRD present the same renal phenotype [51]. Of note, CDK is found in other ciliopathies and typically in the lethal Meckel syndrome [52,53].

### Hepatic features

A minority of JSRD patients present liver disease, usually manifesting as congenital hepatic fibrosis (CHF). This results from an embryonic malformation of the ductal plate, with cystic dilatation of primitive biliary structures and fibrous enlargement of the portal tracts [54]. Liver disease may present with raised serum liver enzymes (alanine aminotransferase, aspartate aminotransferase and gamma-glutamyl transpeptidase) at least twice the normal values, early onset hepato(spleno)megaly or more severe manifestations including portal hypertension, esophageal varices and liver cirrhosis [47,55].

The association of JS with CHF was previously known by the acronym COACH (Cerebellar vermis hypoplasia, Oligophrenia, Ataxia, Coloboma and Hepatic fibrosis) [55,56].

### Skeletal features

Since the description of the first JS family, polydactyly has been often reported in JSRD with a frequency of about 8-16% [1,18,57].

The most common form is represented by postaxial polydactyly, variably affecting hands and feet [58]. The association of JS with polydactyly and oral defects defines a condition known as orofaciodigital type VI or Varadi-Papp syndrome (OMIM 277170) [59]. In this case polydactyly is typically mesaxial with Y-shaped metacarpals.

Mild to severe scoliosis may represent a manifestation of JSRD and likely relates to the degree of hypotonia in early infancy, while structural anomalies of the vertebrae are uncommon [60].

### Miscellaneous

Although JSRD are not typically dysmorphic syndromes, patients have a characteristic face, and a recent study has outlined the presence of peculiar craniofacial features and distinct anthropometric facial patterns, which tend to change with age [20,61]. Congenital heart defects are not typically associated with JSRD but have been reported occasionally [62]. Laterality defects such as complete *situs inversus *are also rare [63]. Hirschsprung's disease has been described so far in two patients with JSRD [64], but its frequency is likely to represent an underestimate. In fact, three individuals with Hirschsprung's disease were observed in a cohort of about 200 patients, suggesting that this association is not coincidental (Brancati and Valente, unpublished observation). Interestingly, Hirschsprung's disease is associated with another ciliopathy, Bardet-Biedl syndrome [65], and cilia have been recently implicated in neural crest development [66].

## Classification of JSRD

The classification system of JSRD is still evolving due to the discovery of novel genes and the improved understanding of genotype-phenotype correlations.

The MTS has been initially described as part of distinct syndromes termed classical JS, JS plus LCA or retinal dystrophy, JS plus polymicrogyria, Senior-Loken, Dekaban-Arima, COACH, Varadi-Papp, and Malta syndromes [4]. Although useful in the past, we now discourage the continued use of such eponyms in favor of a more practical, clinical-genetic classification. The one proposed here defines six subgroups based on the main organ(s) involvement and the established genotype-phenotype correlates (Table 1). Additional features such as facial dysmorphism, polydactyly, colobomas, and other CNS malformations (e.g. encephalocele, abnormal neuronal migration, etc.) can be detected within all the following subgroups.

**Table 1:** Classification of Joubert syndrome and related disorders based on associated clinical features

Clinical subtypes	Mandatory features	Preferentially associated features*	Previously used nosology	Major gene(s)**
Pure Joubert syndrome (JS)	MTS		JS	Mutations in many genes
			JS type A	

JS with ocular defect (JS-O)	MTS			
	Retinal dystrophy (including LCA)		JS type B	*AHI1*

JS with renal defect (JS-R)	MTS			*NPHP1*
	NPH			*RPGRIP1L*

	MTS		Cerebellooculorenal s.	
JS with oculorenal defects (JS-OR)	Retinal dystrophy (often LCA)	(CHF reported in few cases)	SLS plus MTS	*CEP290*
	NPH		JS type B	
			Dekaban-Arima s.	

JS with hepatic defect (JS-H)	MTS	Colobomas	COACH s.	*TMEM67*
	CHF	NPH	Gentile s.	

JS with orofaciodigital defects (JS-OFD)	MTS		Váradi-Papp s.	
	Lobulated/bifid tongue (incl. hamartomas)	Cleft lip/palate	Orofaciodigital VI s.	*TMEM216* (2 patients only)
	Polydactyly			

*Features not correlated with a specific clinical subtype include: postaxial polydactyly; encephalocele and other central nervous system malformations; neuronal migration defects (e.g. polymicrogyria); Hirschsprung's disease; congenital heart defects; laterality defects.

**Besides the major genes listed here, other genes have been occasionally associated with clinical subtypes. Mutations in the *CC2D2A *gene give rise to a wide phenotypic spectrum [13,73,85]. For *INPP5E*, *ARL13B *and *OFD1 *genes, only few mutated patients have been described to date [68-70]. Abbreviations: CHF: congenital hepatic fibrosis; incl.: including; JS: Joubert syndrome; LCA: Leber congenital amaurosis; MTS: molar tooth sign; NPH: nephronophthisis; s.: syndrome; SLS: Senior-Löken syndrome.

### Pure JS

In addition to the MTS, patients display the cardinal neurological findings of hypotonia/ataxia and developmental delay, variably associated with irregular breathing, abnormal eye movements and intellectual disability. There is no retinal, renal or liver involvement. No major gene has been associated with this phenotype, but occasional mutations in several genes have been reported [13,15,67-73].

### JS with ocular defect (JS-O)

The neurological features of JS are present in association to retinal dystrophy (including LCA) with variable age at onset, progression and severity. To date, the most frequently mutated gene in this subgroup is *AHI1*, which accounts for about 20% of cases [39,71,74,75].

### JS with renal defect (JS-R)

In this subgroup, neurological signs are associated with renal disease, which is in most cases juvenile NPH, in the absence of retinal involvement. The two genes most commonly mutated in this rare phenotype are *NPHP1 *and *RPGRIP1L *[11,76-80].

### JS with oculorenal defects (JS-OR)

This form is characterized by the association of neurological signs of JS with both retinal dystrophy and NPH. About 50% of patients carry mutations in the *CEP290 *gene [10,81-83].

### JS with hepatic defect (JS-H)

This subgroup presents the association of JS with CHF. Chorioretinal or optic nerve colobomas and NPH can be part of the phenotype but are not mandatory features. Over 70% of cases are due to mutations in the *TMEM67 *gene [14,47,72,84,85].

### JS with oro-facio-digital defects (JS-OFD)

In this subgroup, JS features are associated to bifid or lobulated tongue (often described as soft-tissue nodules or multiple hamartomas), multiple oral frenulae and polydactyly, that is usually mesaxial, with Y-shaped metacarpals [4,59,86,87]. Hypothalamic hamartoma or congenital absence of the pituitary gland can be part of this spectrum [38,86,88]. This phenotype has been recently associated with mutations in the *TMEM216 *gene [15].

## Diagnosis

**Figure 2:**
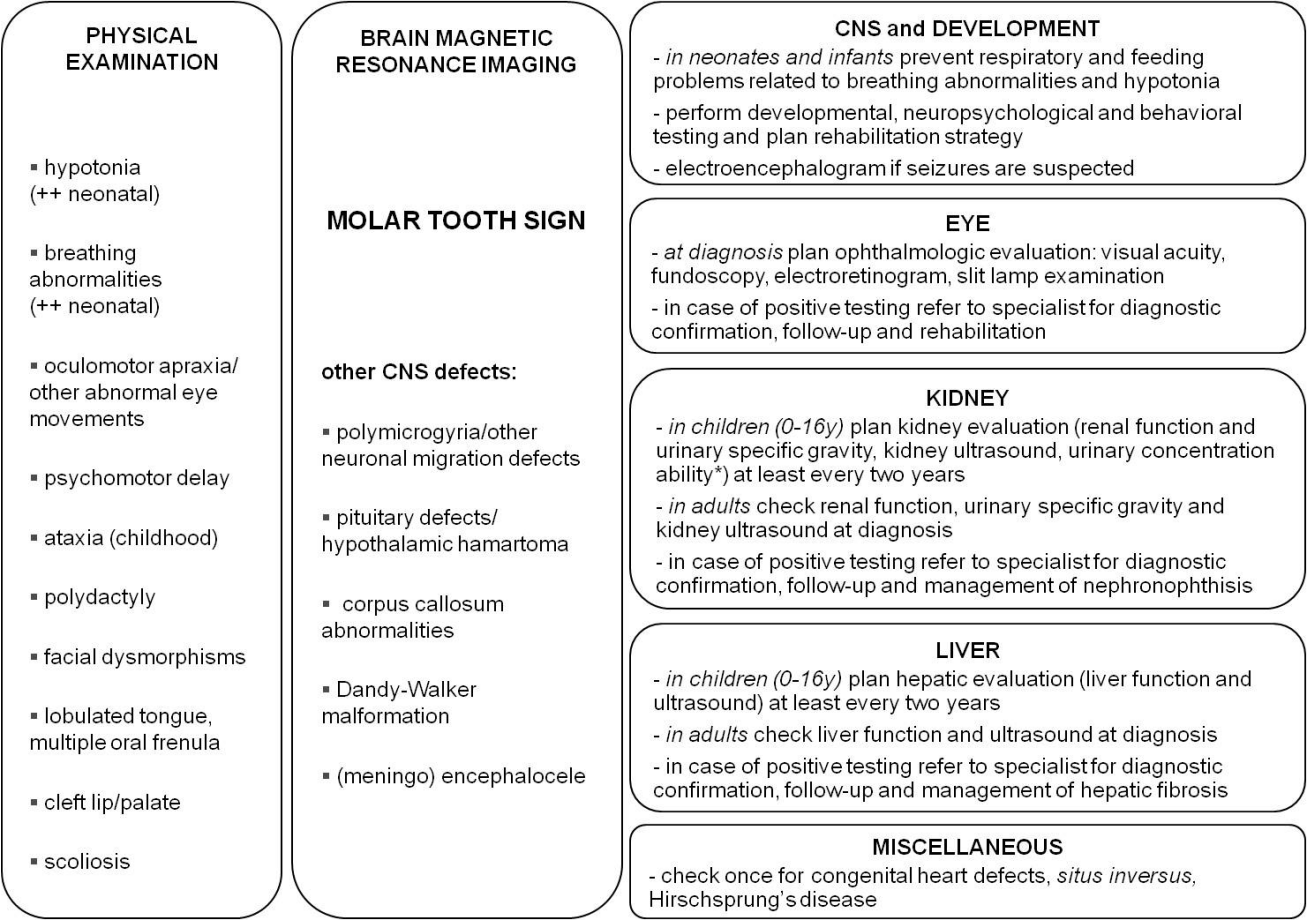
**Schematic approach to the diagnosis and management of JSRD**. Left: clinical manifestations that - in variable association - may lead to the diagnostic suspicion of JSRD. Middle: the detection of the MTS by brain magnetic resonance imaging confirms the diagnosis; in a subset of patients, other CNS defects may be identified. Right: proposed evaluation protocol for multiorgan involvement. *by water restriction or Desmopressin test.

A diagnosis of JSRD should be suspected in all infants presenting with hypotonia, abnormal eye movements (in particular oculomotor apraxia, but also nystagmus) and developmental delay. The occurrence of abnormalities in the respiratory pattern, i.e. hyperpneas alternating with periods of apnea, reinforces the clinical suspicion of the disease. In these children, a brain MRI is sufficient to confirm or exclude the diagnosis, based on the detection of the MTS (Figure 2).

Once a diagnosis of JSRD has been made, children should enter a diagnostic protocol to assess the possible multiorgan involvement. Ocular investigations include evaluation of visual acuity, ocular motility, *fundus oculi *and, whenever possible, electroretinogram. Slit lamp examination can disclose abnormalities of the anterior segment of the eye. Kidney and liver function should be tested. Standard urine analysis is also necessary, and special attention should be given to urine specific gravity; should this value be abnormal or towards the lower normal range, a challenge test to assess urinary concentration ability is recommended. This is usually performed with water restriction or, even better, by evaluating urinary specific gravity after stimulation with Desmopressin. Abdominal ultrasound will explore the kidneys (to detect small cysts and loss of corticomedullar differentiation, suggestive of NPH), and the liver (to identify hepatomegaly or structural abnormalities that could underlie CHF). If hepatic involvement is suspected, further liver imaging techniques (such as magnetic resonance imaging) can support the diagnosis by demonstrating bile ducts proliferation or fibrotic nodules. Diagnosis is then confirmed by liver biopsy.

Possibly associated signs need to be investigated, including pituitary defects, cleft palate, lobulated tongue, congenital heart defects, *situs inversus *and Hirschsprung disease. A careful assessment of brain imaging is also requested to search for associated CNS malformations (Figure 2).

To date, diagnostic genetic testing is available only for a few genes, while selected laboratories offer molecular testing of known genes on a research basis. Genes to be tested should be prioritized by taking into account the clinical subgroup to which the patient belongs and the available genotype-phenotype correlates (Table 1).

## Differential diagnosis

All conditions displaying neurological (hypotonia, abnormal breathing, abnormal eye movements and developmental delay) and/or organs manifestations seen in JSRD (retinal dystrophy, NPH, CHF, polydactyly) should be considered. In particular, these include other ciliopathies such as isolated nephronophthisis, Senior-Loken syndrome and Bardet Biedl syndrome, as well as cerebellar and brainstem congenital malformations and cerebro-oculo-renal syndromes [48,89-91].

## Genetic counseling and prenatal diagnosis

With the exception of rare cases following X-linked recessive inheritance [70], JSRD are transmitted in autosomal recessive fashion, and the recurrence risk for a couple with an affected child is one in four. Prenatal diagnosis is feasible through chorionic villus sampling at around eleven weeks gestation, only in families in which the molecular defect had been previously identified in the proband. To date, published mutation screenings of known genes have allowed the identification of mutations in less than half JSRD patients, making prenatal diagnosis still limited to a subset of families [92].

In the remaining families, fetal ultrasound may be useful in at-risk pregnancies, allowing the detection of hypoplasia of the cerebellar vermis and, when present, occipital encephalocele. Polydactyly may also represent a suggestive, although non-specific, feature possibly associated with all JSRD phenotypes [93,94]. Recently, fetal MRI has been acknowledged as the method of choice to delineate posterior fossa malformations, facilitating the diagnosis of the disease before 24 weeks of gestation [95-97].

## Management and follow-up

In neonates and infants particular care should be taken in managing respiratory and feeding problems related to either breathing abnormalities or hypotonia. Rehabilitation strategies must be planned for cognitive and behavioral difficulties and specific manifestations such as the visual impairment.

Any abnormal feature identified during the diagnostic assessment should be carefully followed up over time. In particular, the detection of decreased urinary concentration ability often represents the first clue to a diagnosis of NPH in otherwise asymptomatic patients. This requires a close monitoring to timely recognize and treat early signs of renal failure and to delay the onset of complications such as growth defect or bone disease. If CHF is diagnosed, specific follow-up should be planned to manage possible complications, including portal hypertension and esophageal varices.

## Prognosis

Soon after birth, prognosis is related to the extent and severity of breathing dysregulation. In particular, recurrent episodes of prolonged apneas can be life-threatening and require assisted ventilation. In most cases, these respiratory abnormalities resolve spontaneously in the first months or years of life. Feeding difficulties may represent a problem in a number of patients. Afterwards, prognosis depends mostly on renal and hepatic complications that, if not timely diagnosed and managed, represent the major causes of death in JSRD patients.

## List of abbreviations used

JS: Joubert syndrome; MTS: molar tooth sign; JSRD: Joubert syndrome and related disorders; CNS: central nervous system; LCA: Leber congenital amaurosis; NPH: nephronophthisis; CDK: cystic dysplastic kidneys; CHF: congenital hepatic fibrosis.

## Competing interests

The authors declare that they have no competing interests.

## Authors' contributions

FB and EMV revised the literature and drafted the manuscript; BD critically revised the manuscript for intellectual content. All authors read and approved the final manuscript.
